# Medical Works

**Published:** 1858

**Authors:** 


### Medical Works.

We have looked over the assortment of Medical books with which Ogden, Brownell & Co., booksellers of this city, have supplied themselves, and find it to contain a choice, as well as a large list of the best works extant. The above firm has exhibited a lively interest in behalf of the profession, in thus putting it into the power of the medical men of this State, and the surrounding country in adjoining States, of procuring at low prices the best standard works, for which the profession should feel deeply grateful. From our examination we would feel assured they can supply every want in this particular, which we esteem a great privilege and accommodation. We are sure they will be well sustained and encouraged to continue to keep themselves supplied so that the physician in the interior will be assured that his orders will be promptly filled.

The firm of Hart & Barber have a large assortment of miscellaneous books, and expect in a few weeks to be supplied with all the late medical works, offering the profession greater inducements to make their purchases in this city.

